# Insights into sepsis-induced apoptosis: Interplay between programmed cell death and interleukin-7

**DOI:** 10.2478/jccm-2025-0003

**Published:** 2025-04-30

**Authors:** Anca Meda Văsiesiu, Oana Coman, Raluca Stefania Fodor, Anca Bacârea, Bianca-Liana Grigorescu

**Affiliations:** George Emil Palade University of Medicine, Pharmacy, Science, and Technology of Targu Mures, Romania

**Keywords:** sepsis, septic shock, PD-1/PD-L1 axis, IL-7, apoptosis, programmed cell death

## Abstract

The pathophysiology of sepsis is orchestrated by a delicate and dynamic interaction between pro-inflammatory and anti-inflammatory responses. Essential factors influencing this process include interleukin-7 (IL-7), the programmed cell death protein 1/programmed death ligand 1 (PD-1/PD-L1) axis, and cellular apoptosis. These elements shape the immune response in sepsis, influencing its progression and outcomes. IL-7 is an important cytokine maintaining lymphocyte function and survival. At the same time, the PD-1/PD-L1 axis acts as a modulatory checkpoint suppressing immune activation to prevent overreaction but can exacerbate immunosuppression during sepsis. Cellular apoptosis impairs the host’s ability to mount an effective defence, especially against secondary infections.

Despite extensive research, the precise mechanisms through which sepsis results in organ dysfunction and immune dysregulation remain incompletely understood. The global burden of sepsis emphasizes the urgent need for innovative approaches, paving the way for personalized, immune-based therapies.

This review aims to delve into and synthesize the current knowledge regarding cellular apoptosis, the regulatory role of the PD-1/PD-L1 axis, and the critical functions of IL-7 in sepsis, with a focus on their underlying mechanisms, clinical relevance, and potential as targets for future immunomodulatory treatments.

## Introduction

Sepsis continues to pose a significant global health challenge and remains a major cause of mortality in intensive care units worldwide. Recognizing its critical impact, in 2020, the World Health Organization has designated sepsis as a Global Health Priority [1,2]. The report emphasizes the significant health burden of sepsis, with an in-hospital mortality rate exceeding one-third among sepsis patients treated in the intensive care units (ICU), peaking at 42% [2]. A 2017 global study estimated 49 million cases of sepsis and 11 million related deaths, accounting for roughly 20% of annual global mortality. The estimated average cost of hospital-based sepsis treatment exceeds $32,000, based primarily on data from economically developed countries [3,4].

Sepsis is characterized by immune dysregulation triggered by various infections. This dysregulation is marked by the coexistence of an excessive inflammatory response and persistent immune suppression [5,6]. The 2023 guidelines redefined sepsis as “a life-threatening organ dysfunction resulting from a dysregulated host response to infection.” This definition highlights the complexity of its pathogenesis, encompassing inflammatory disruption, changes in immune response, impaired microcirculation, mitochondrial injury, and coagulation abnormalities, ultimately resulting in severe circulatory and metabolic disturbances [7,8].

The traditional biphasic concept, which proposed that an initial hyperinflammatory phase is followed by a subsequent immunosuppressive phase, is no longer considered accurate [1]. Despite this, the pathogenesis of sepsis involves two overlapping phases—hyperinflammation and immunoparalysis—both emerging at the onset of the condition and contributing to widespread organ failure [1].

Critically ill patients often experience organ dysfunction, frequently resulting from a dysregulated inflammatory host response. Immune mechanisms are pivotal in the progression of organ dysfunction in these patients [9]. Beyond the impact of an overactive innate immune response, there is growing evidence linking the vulnerability of critically ill patients to secondary infections with an impaired immune response, regardless of the primary insult. Modulating the host response holds the theoretical potential to accelerate the resolution of organ injury and provide protection against secondary infections [10].

Although the patient’s immune responses vary, immune paralysis emerges early in sepsis and is an integral factor in the progression to multiple organ failure [11].

The immune suppression observed in sepsis hinders the body’s ability to clear the primary infection and significantly raises the risk of secondary infections. Crucially, the persistent immunosuppressive state, driven by impaired innate and adaptive immune mechanisms, contributes to weakened immunity, multi-organ failure, extended hospital stays, and elevated mortality rates. A comprehensive understanding of the mechanisms underlying sepsis-induced immunosuppression is imperative to understand this condition effectively [12,13]. In this light, sepsis’ pathophysiology remains a “Pandora’s box” comprising multiple and unveiling facets of hidden fine-tuned immune mechanisms.

Although advances in sepsis management, including antimicrobial therapies and fluid therapy, have helped lower mortality rates in recent decades, significant opportunities for further improvement remain.

The mechanisms through which sepsis causes organ dysfunction remain incompletely understood, representing a critical gap in light of the persistently high mortality rates, the limited and non-specific therapeutic options, and the significant long-term morbidity faced by survivors [14].

Sepsis has a profound impact and demands the development of innovative approaches to address it [15]. Advancements in understanding the immunomodulatory processes present opportunities to develop personalized immune-based therapies.

This review seeks to examine and consolidate the existing understanding of lymphocyte apoptosis, the involvement of the programmed cell death protein 1/programmed death ligand 1 (PD-1/PD-L1) axis, and interleukin-7 (IL-7) in sepsis, with an emphasis on their underlying processes, clinical significance, and potential for future immunomodulatory treatments.

## Apoptosis and immunoparalysis in sepsis

In sepsis, initial immune response to pathogens is triggered by the activation of innate immune cells, such as neutrophils, macrophages, monocytes, and natural killer (NK) cells. This process begins with the recognition of pathogen-associated molecular patterns through specific pattern recognition receptors. [16,17,18]. These interactions trigger intracellular signal transduction pathways, leading to the activation of transcription and the subsequent release of proinflammatory cytokines such as tumour necrosis factor-α (TNF-α), IL-1, and IL-6 [17,18].

A prolonged state of immunosuppression follows the initial proinflammatory response in sepsis. During this phase, T cell numbers, including both helper and cytotoxic T cells, decrease due to apoptosis, leading to a reduced response to inflammatory cytokines. Post-mortem studies of septic patients revealed significant depletion of CD4+ and CD8+ T cells, primarily in lymphoid organs such as the spleen. Further research has also demonstrated a reduction in the levels of key cytokines like IL-6 and TNF in response to endotoxins [17].

## Innate immune system

Apoptosis ensues in tissue cells and leukocytes that are no longer functional at the site of infection. These apoptotic cells stimulate the production of IL-10 and transform growth factor β. Moreover, lipoxygenin, a bioactive lipid, can induce apoptosis. Sepsis triggers delayed apoptosis of neutrophils originating from bone marrow, leading to a rapid increase in bloodstream neutrophil levels. Ongoing neutrophil dysfunction, along with the emergence of immature neutrophils, results in neutrophil deficiency [19]. Delayed neutrophil apoptosis and increased apoptosis of other immune cells weaken the host immune system, inducing detrimental effects that extend beyond the substantial loss of immune cells [19, 20].

Dendritic cells (DCs) are essential for activating adaptive immunity. Their apoptosis and reduction in secondary lymphoid organs increase the organism’s risk of secondary infections. Moreover, DCs become immunoparalysed and fail to activate T lymphocytes, leading to a septic homeostatic imbalance and a reduced secretion of IL-12. Conversely, IL-12 is essential for the interplay between immunoparalysed DCs and CD8+ cells originating from septic bone marrow. The function of immunological tolerance and outcomes in ICU patients is linked to the highly expressed transcription factor B lymphocyte-induced maturation protein on DCs. The loss of this function compromises the responsiveness of T cells to infection [20].

NK lymphocytes and cytotoxic T lymphocytes are regarded as essential elements of the cytotoxic cell axis and play a pivotal role in the progression of septic shock. A defining characteristic of NK cells is their absence of specific T-cell receptors, resulting in a non-specific immune response that allows NK cells to target intracellular pathogen invasion [20,21].

In Gram-negative bacteria sepsis, the number of circulating NK cells was significantly lowered and was associated with increased mortality [7]. Extensive sepsis-induced apoptosis of natural killer T (NKT) lymphocytes is a leading cause of immunosuppression, but their underlying regulatory mechanisms in sepsis remain evasive [22].

The hyperinflammatory phase results in abnormalities in NK cell activation, cytokine storms driven by a positive feedback loop, and extensive organ impairment. Cytotoxic dysfunction and reduced levels of interferon-γ (IFN) in NK cells make post-septic patients susceptible to secondary infections [20]. The immunosuppressive phase of sepsis is marked by a NK dysfunction and an increased tendency to apoptosis [7]. The therapeutic administration of a homogeneous group of cells or multiple cytokines, such as IL-2, IL-12, or IL-18, during the immunosuppressive phase of sepsis, could enhance the patient’s outcome [20].

## Adaptative immune system

In sepsis and septic shock, lymphopenia is a consequence of extensive apoptosis and could serve as a predictor of both early and late mortality. T cells are particularly susceptible to apoptosis and functional suppression, leading to profound lymphopenia [23]. Even if the absolute number of T cells may recover, significant phenotypic and functional changes occur: altered cytokine production, increased expression of multiple inhibitory receptors, and a decrease in the regenerating capacity of memory T cells [20].

In sepsis survivors, memory T cells are immunoparalysed due to secondary infections, but memory T cell compartments are altered. Various subtypes of irregular T cells, such as gamma-delta (γδ) T cells, mucosal-associated invariant T cells, and NKT cells, have key roles in sepsis immunology [20].

Sepsis is associated with apoptosis and functional changes in immune cells, including CD4+ and CD8+ T cells, B cells, and dendritic cells. CD4+ T helper (Th) cells, essential for a proper immune response, are classified into several subtypes: Th1, Th2, and Th17 effector T (Teff) cells, along with distinct subpopulations such as regulatory T cells (Tregs) and Th9 cells [23].

Previous studies have shown that a persistently elevated Th2/Th1 ratio is associated with the greatest 28-day mortality and a higher rate of ICU-acquired infections [20].

In sepsis, Tregs increase and suppress the functionality of Teff cells, monocytes, and neutrophils, leading to immunoparalysis. Continuous stimulation of T cells leads to their exhaustion and loss of their effector function [23].

NK lymphocytes and cytotoxic T lymphocytes are components of the cytotoxic cell axis, and are vital in the progression of septic shock in patients, though their dynamic is not fully understood. Numerous studies revealed that circulating CD8+ T and NK cells are significantly decreased in patients with septic shock but increased in the spleen and lymph nodes [21].

Studies have shown that the reduction of circulating CD8+ T and NK cells in septic shock patients triggers a counterbalancing increase in activated CD8+ T and NK cells to defend against the invasion of pathogenic bacteria. In non-survivors, a significant amount of the activated CD8+ T cells were consumed in large quantities, suppressing their killing function [21]. This could be explained by the important role played by CD8+ T cells in viral infections, but they present a lesser role in bacterial sepsis [24].

A study published by Chen et al. highlights that the immune cell count, or their apoptosis level, cannot reflect the immune status in sepsis. The metabolic activity, cytotoxic function, and expression of apoptosis-related receptors in CD4+ and CD8+ T cells can serve as indicators of T cell immune status. Anomalous upregulation of metabolic and apoptotic receptors on CD4+ T cells, along with a reduction in functional factors, are predictive of poor outcomes in critically ill septic patients [24].

In septic shock patients, B cells are decreased in peripheral blood. The decrease of immature, naïve, and resting memory B cells leads to an increased percentage of memory B cells and activated memory B cells. In sepsis, regulatory B cells are increased in peripheral blood and are involved in the negative regulation of innate immune response by increasing their secretion of anti-inflammatory cytokines and by promoting Treg cell response. Extensive apoptosis of B cells and the amplification of their suppressive subpopulation are the key points to the altered immune response in sepsis [20].

## The immunosuppressive role of PD-1/PD-L1 axis in sepsis

Intrinsic to the immune system, immune checkpoint pathways regulate immune responses under normal physiological conditions. Among these pathways, the programmed cell death protein 1 (PD-1) and its ligand, programmed death ligand 1 (PD-L1), serve as key regulators by inhibiting T cell receptor-mediated activation signals. Additionally, the interaction between PD-1 and PD-L1 leads to systemic immune suppression across various cell types [25,26].

PD-1, also referred to as CD279, is a co-inhibitory receptor initially identified on antigen-activated T lymphocytes. Expression of PD-1 has also been observed in a small subset of cells in lymph nodes, the spleen, bone marrow, and immature CD4+CD8+ thymocytes. Its expression is often upregulated following the activation of B cell receptors or T cell receptors, highlighting its role in modulating immune responses [27,28]. The expression of PD-1 on various cell types is influenced by multiple regulatory mechanisms. PD-1 levels expressed on T lymphocytes increase after antigen activation. If the antigen is efficiently cleared, PD-1 expression decreases on the responding T cells; however, persistent antigen presence sustains elevated PD-1 levels [29,30]. Key regulators of PD-1 expression in T cells include nuclear factor of activated T cells, Forkhead Box Protein O1, T-bet, B lymphocyte-induced maturation protein 1, and glycogen synthetase kinase 3. T cell receptor activation serves as the primary driver of PD-1 expression in these cells [5, 31].

PD-L1 (CD274) and PD-L2 (CD273) are the two known ligands for PD-1. PD-L1 is extensively expressed on hematopoietic cells, including T and B lymphocytes, macrophages, and DCs, along with non-hematopoietic cells such as vascular endothelial cells, keratinocytes, pancreatic islet cells, astrocytes, corneal epithelial cells, and other healthy tissue cells [13]. In contrast, PD-L2 expression is more limited, being primarily observed on macrophages, DCs, and mast cells. The primary mechanism by which PD-1 functions in sepsis involves its interaction with PD-L1. Notably, PD-L1 gene deficiency has been shown to improve survival outcomes in sepsis, whereas PD-L2 gene deficiency does not confer the same survival advantage [32,33].

T cell activation is suppressed by PD-1 expression through the phosphorylation of the immunoreceptor tyrosine-based switch motif, which triggers downstream effector molecules that block T cell proliferation and cytokine production [9]. Studies of postmortem tissues from septic patients have identified profound dysfunction in spleen cells, accompanied by immunosuppressive effects in the lungs. These effects may result from immune cell death triggered by self-programmed mechanisms during immunosuppression [17,35]. Chronic antigen stimulation in sepsis leads to the upregulation of inhibitory receptors on T cells, a key mechanism driving immunosuppression. PD-1 expression in T cells is induced by T cell receptor signalling and cytokines like IL-2, IL-7, and type I IFNs. Notably, this expression is heightened in activated T cells and can be modulated within 24 hours based on the intensity or concentration of the stimulus. Its inhibitory effects are driven by interactions with the ligands PD-L1 and PD-L2 [36].

Research has demonstrated that elevated expression of PD-1 and PD-L1 on T cells, monocytes, and neutrophils are closely associated with immunosuppression in sepsis, making it a significant risk factor for mortality among septic patients. This insight into sepsis-induced immunosuppression offers valuable perspectives on its pathophysiology and highlights a promising avenue for developing targeted therapies and improving prognostic predictions [37].

Recent animal studies utilizing the classic cecal ligation and puncture-induced sepsis mouse model have revealed that sepsis begins at an early stage, marked by apoptosis of splenocytes, a reduction in CD4+ and CD8+ T cells, and elevated PD-L1 expression on myeloid-derived suppressor cells (MDSCs). Among these, polymorphonuclear-MDSCs represent the predominant subset and exert significant immunosuppressive effects through the PD-L1/PD-1 axis during the early phases of sepsis [38].

In sepsis, human immune cells exhibit elevated expression of PD-1 and related molecules. Notably, PD-1 levels increase on CD4+ T cells in septic patients, and this upregulation is strongly associated with worse clinical outcomes and poor prognosis [24]. In septic patients, NK cells show a marked increase in PD-L1 expression, with its prevalence serving as an independent predictor of 28-day mortality. Even after sepsis recovery, soluble PD-L1 levels remain elevated in the bloodstream, correlating with higher readmission rates and overall mortality over a six-month period. Furthermore, PD-1/PD-L1 axis expression is also elevated across distinct subsets of memory B cells and T cells in individuals with sepsis, underscoring the immune system’s persistent dysregulation [37, 40,41].

## IL-7 an anti-apoptotic and immune cells’ restoring factor

Lymphocyte depletion in sepsis is closely associated with lymphopenia, characterized by a marked reduction in their absolute circulating number. Lymphocytes play a crucial role in mounting an effective immune response against invading pathogens, making their depletion a critical concern in septic conditions [42,43]. Research conducted by various independent laboratories has demonstrated that mice with lymphocytes resistant to sepsis-induced apoptosis exhibit improved survival rates. This resistance is achieved either by overexpressing the antiapoptotic protein Bcl-2 or by deleting the pro-apoptotic protein Bim [43,44].

Interleukin-7 (IL-7) is a globular protein produced by various cell types, including fetal liver cells, stromal cells within the bone marrow and thymus, as well as keratinocytes and enterocytes [45]. It is a potent antiapoptotic cytokine that promotes lymphocyte survival by inducing the expression of Bcl-2 and supporting the proliferation of CD4+ and CD8+ T cells [46]. Its protective effects extend beyond preventing apoptosis; IL-7 enhances lymphocyte expansion and function. Multiple studies have demonstrated that IL-7 not only prevents lymphocyte apoptosis but also restores the function of CD4+ and CD8+ T cells, leading to improved survival outcomes in animal models of bacterial and fungal sepsis. Additionally, ex vivo research on peripheral blood from patients with septic shock has shown that IL-7 improves T cell cytokine production, restores lymphocyte metabolism, and counters lymphocyte apoptosis [47].

The IL-7 receptor (IL-7R) is a heterodimeric complex comprising the α-chain and the common γ-chain, a component shared with receptors for cytokines like IL-2, IL-4, IL-9, IL-15, and IL-21. This receptor is expressed on various cell types, enabling IL-7 to exert diverse biological effects. Through its interaction with IL-7R, IL-7 influences various cell types. Deficiencies in either IL-7 or IL-7R can result in profoundly disrupted immune cell development, highlighting its critical role in immune function [48,49].

IL-7 contributes significantly to the progression and management of sepsis. Acting as a critical antiapoptotic factor, IL-7 supports both lymphocyte survival and expansion while also stimulating CD4+ and CD8+ T cell proliferation [43]. Beyond maintaining peripheral T cell homeostasis, increased IL-7 production also supports the survival of both naïve and memory T cells. In addition, IL-7 is involved in multiple stages of B-cell progenitor development, including commitment, survival, differentiation, and proliferation. Moreover, IL-7 is an essential cytokine with a distinct role in recruiting immune cells such as neutrophils and monocytes, highlighting its multifaceted contributions to immune regulation [50,51]. Also, research has found that lower IL-7 levels are associated with increased severity and mortality in critically ill patients with COVID-19, further underscoring its importance in immune modulation [52].

For critically ill patients admitted to the ICU, determining the immune system’s precise response timing to infection is challenging. Although the immune system typically activates to restore homeostasis and ensure proper T lymphocyte function, some patients fail to adequately increase IL-7 synthesis. This failure can result in higher mortality rates. Low IL-7 levels may stem from either impaired activation of the IL-7 signalling pathway or delays in its activation, undermining the immune response’s effectiveness [9, 53].

IL-7 signalling is necessary for T cell development; deficiencies in IL-7 or its receptor in humans resulting in significantly compromised T lymphopoiesis [49]. IL-7 is essential for normal thymopoiesis, and while IL-7 therapy in aged mice has not reversed thymic involution, studies suggest it can gradually enhance thymic recovery [55]. The regulation of IL-7R expression during T cell development is paramount, as IL-7R is essential for the transition of signalling double-positive thymocytes into functional CD8+ T cells [49,56].

IL-7 enhances B-cell survival by influencing the balance of pro-apoptotic proteins (e.g., Bax, Bad, and Bim) and antiapoptotic factors (e.g., Bcl-2, Bcl-xL, and Mcl-1). Mature B cells typically show little response to IL-7 due to low IL-7R expression; however, elevated IL-7 levels can still promote B-cell survival and enhance antibody production in the presence of T cells, even without additional B-cell stimulatory signals. This process occurs through IL-7-mediated activation of CD4+ T cells, which boosts CD70 expression on memory cells, thereby aiding in B-cell activation. Furthermore, IL-7 treatment prompts resting peripheral T cells to release B-cell activating factors, further promoting B-cell survival [49,57].

Sepsis typically progresses through an initial hyperinflammatory phase, leading to organ dysfunction and early mortality, followed by a prolonged immunosuppressive phase. This later stage is associated with an increased risk of secondary infections and delayed mortality. The immunosuppressive state is marked by immune dysfunctions, including impaired antigen presentation and altered lymphocyte subsets. Notably, this phase is characterized by a sustained T cell exhaustion phenotype, evident in reduced T cell counts, diminished T lymphocyte functionality, a higher proportion of Tregs, and elevated expression of co-inhibitory molecules [54,58].

Developing innovative therapeutic strategies to address the immunosuppressive state induced by sepsis is of paramount significance. Research into immunoadjuvant therapies addressing adaptive immunity defects in sepsis patients has gained momentum in recent years. CYT107, a glycosylated recombinant human IL-7, has shown promise when administered intramuscularly. Beyond boosting circulating lymphocyte numbers, CYT107 enhanced lymphocyte activation, indicating its potential to mitigate the T cell dysfunction associated with sepsis-induced immunosuppression [43,59].

## Conclusions

Despite years of research and significant progress in uncovering the precise pathophysiological mechanisms of sepsis, it continues to pose a major challenge for clinicians. The intricate processes underlying the initial hyperinflammatory phase and subsequent immunosuppression are attributed to the interplay of three key pillars: cellular apoptosis, secondary immunosuppression driven by the activation of the PD-1/PD-L1 axis, and IL-7, often regarded as the “master regulator” of the immune system. A deeper understanding of these mechanisms paves the way for innovative immunomodulatory therapies and the development of personalized treatment strategies.
